# Phospholipase PLA2G7, associated with aggressive prostate cancer, promotes prostate cancer cell migration and invasion and is inhibited by statins

**DOI:** 10.18632/oncotarget.397

**Published:** 2011-12-22

**Authors:** Paula Vainio, Laura Lehtinen, Tuomas Mirtti, Mika Hilvo, Tuulikki Seppänen-Laakso, Johannes Virtanen, Anna Sankila, Stig Nordling, Johan Lundin, Antti Rannikko, Matej Orešič, Olli Kallioniemi, Kristiina Iljin

**Affiliations:** ^1^ Medical Biotechnology, VTT Technical Research Centre of Finland, and Turku Centre for Biotechnology, University of Turku, Finland; ^2^ Haartman Institute, Department of Pathology, University of Helsinki, Finland; ^3^ Institute for Molecular Medicine Finland (FIMM), University of Helsinki, Finland; ^4^ HUSLAB, Department of Pathology, Helsinki University Central Hospital, Finland; ^5^ Bio and Process Technology, VTT Technical Research Centre of Finland, Espoo, Finland; ^6^ Department of Urology, Helsinki University Central Hospital, Finland

**Keywords:** Prostate cancer, PLA2G7, drug target, biomarker, statins

## Abstract

Prostate cancer is the second leading cause of cancer mortality in men in developed countries. Due to the heterogeneous nature of the disease, design of novel personalized treatments is required to achieve efficient therapeutic responses. We have recently identified phospholipase 2 group VII (*PLA2G7*) as a potential drug target especially in ERG oncogene positive prostate cancers. Here, the expression profile of PLA2G7 was studied in 1137 prostate cancer and 409 adjacent non-malignant prostate tissues using immunohistochemistry to validate its biomarker potential and putative association with disease progression. In order to reveal the molecular alterations induced by *PLA2G7* impairment, lipidomic and gene expression profiling was performed in response to *PLA2G7* silencing in cultured prostate cancer cells. Moreover, the antineoplastic effect of statins combined with PLA2G7 impairment was studied in prostate cancer cells to evaluate the potential of repositioning of *in vivo* compatible drugs developed for other indications towards anti-cancer purposes. The results indicated that PLA2G7 is a cancer-selective biomarker in 50% of prostate cancers and associates with aggressive disease. The alterations induced by *PLA2G7* silencing highlighted the potential of PLA2G7 inhibition as an anti-proliferative, pro-apoptotic and anti-migratorial therapeutic approach in prostate cancer. Moreover, the anti-proliferative effect of *PLA2G7* silencing was potentiated by lipid-lowering statins in prostate cancer cells. Taken together, our results support the potential of PLA2G7 as a biomarker and a drug target in prostate cancer and present a rationale for combining PLA2G7 inhibition with the use of statins in prostate cancer management.

## INTRODUCTION

Although prostate cancer is the most commonly diagnosed malignancy and the second most common cause of cancer mortality in men in developed countries [1], there is a prevailing lack of efficient targeted and personalized therapeutic approaches. Given the heterogeneous nature and the complexity of molecular pathways in prostate cancer, combining different therapies may be a necessary step towards significant therapeutic progress.

Approximately half of prostate cancer samples harbor an oncogenic gene fusion combining androgen regulated transmembrane protease serine 2 (*TMPRSS2*) with oncogenic ETS transcription factors [2]. Ectopic expression of the most frequent fusion partner, *ERG* (v-ets erythroblastosis virus E26 oncogene homolog, avian), promotes multiple signaling pathways associated with cancer formation and progression [3-7]. However, ETS gene fusions are a challenge to target and *ERG* mediated oncogenic processes may be bypassed in advanced prostate cancer [8]. Therefore, novel more efficient therapeutic approaches for this patient group, as well as for the early disease, would be of great importance.

Phospholipase A2 group VII (*PLA2G7*) was recently found to be highly expressed especially in the tumors with high ERG expression [4, 9]. *ERG* was shown to induce the expression of *PLA2G7*, and knock-down of *PLA2G7* significantly reduced the growth of ERG positive, but not ERG negative, prostate cancer cells *in vitro*, indicating potential as a biomarker and personalized drug target in ERG positive prostate cancers [9]. Furthermore, *PLA2G7* silencing was shown to sensitize prostate cancer cells to oxidative stress [9]. However, the molecular alterations in response to *PLA2G7* expression in prostate cancer remain to be elucidated.

In contrast to cancer, the role and therapeutic potential of PLA2G7 has been under intensive research in the area of cardiovascular diseases. Although PLA2G7 has been shown to exert anti-inflammatory effects in a variety of experimental models, it also degrades apoptosis inducing oxidized phospholipids and simultaneously generates atherogenic inflammatory products [10-12]. Accordingly, PLA2G7 mass and activity have been associated with an increased risk of cardiovascular diseases [13-16]. Interestingly, early results with PLA2G7 inhibitor, darapladib, have been promising in the prevention and treatment of coronary heart disease [11, 17]. In addition, lipid-lowering statins are known to reduce PLA2G7 mass and activity in plasma and atherosclerotic plaques [14, 18, 19].

The aim of this study is to validate PLA2G7 as potential cancer selective biomarker, deepen our understanding on its molecular and cellular function and study the growth inhibitory potential of PLA2G7 impairment combined with statin exposure in cultured prostate cancer cells. PLA2G7 expression was studied in a large set of non-malignant prostate and prostate cancer tissues using immunohistochemistry. In order to reveal the changes induced by PLA2G7 impairment in prostate cancer cells, lipidomic and gene expression profiling was performed in cultured prostate cancer cells. The antineoplastic effect of statins combined with PLA2G7 impairment was studied in prostate cancer cells to evaluate the potential for repositioning of *in vivo* compatible drugs developed for other indications towards anti-cancer purposes.

## RESULTS

### PLA2G7 is a potent biomarker distinguishing prostate cancer from non-malignant prostate tissues

**Figure 1:**
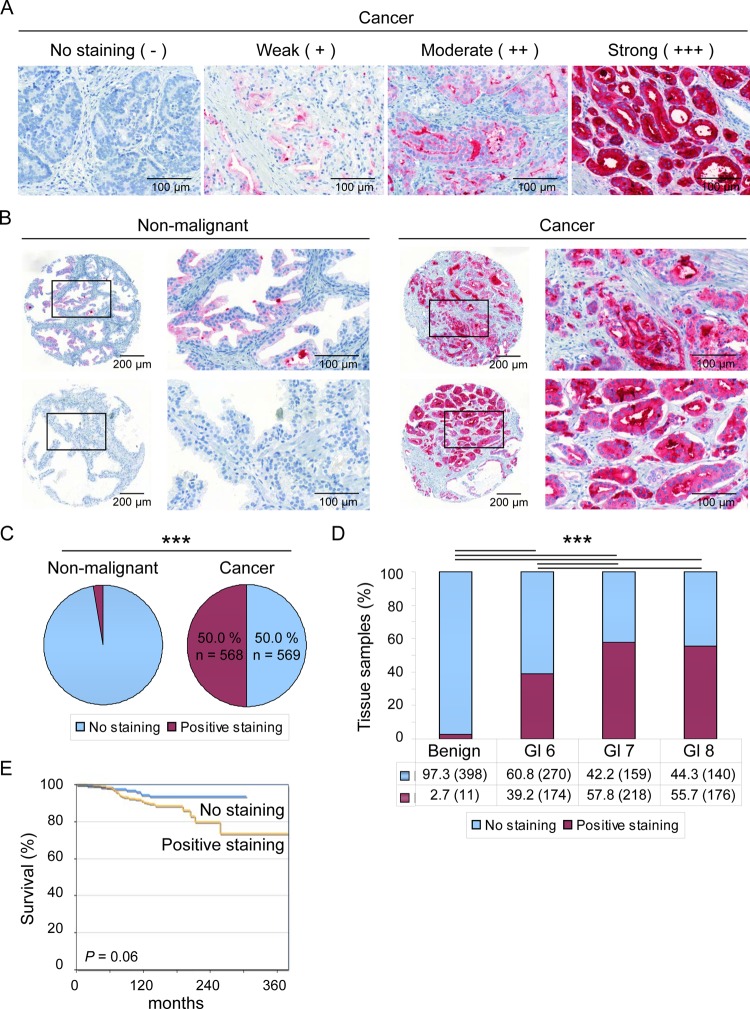
PLA2G7 is expressed in a cancer specific manner and associates with aggressive disease (A) Staining intensity of PLA2G7 in primary prostate cancer (n = 1,137) and adjacent non-malignant prostate (n = 409) samples was scored as follows: Strong (+++), moderate (++), weak (+) or no staining (negative). Representative section of each staining intensity is presented. (B) Immunohistochemical staining results of PLA2G7 expression in adjacent non-malignant and cancer tissue samples for two patients are shown. The areas presented at higher magnification have been indicated in the core images. (C) The proportion of tissue samples with no staining and positive PLA2G7 staining in non-malignant and cancer tissue samples included in the TMA. (D) The proportion of TMA tissue samples with no PLA2G7 staining or positive PLA2G7 staining in non-malignant and cancer tissue samples according to Gleason score. The amount of samples in each group is indicated in parentheses. Significant p-values between different histological stages are presented. (E) Kaplan-Meier curve presentation of prostate cancer specific survival in the patient groups with no PLA2G7 staining (n = 135) or positive PLA2G7 staining (n = 230) in the cancer samples.

Tissue microarray (TMA) containing samples from primary prostate tumors (n = 1137) along with adjacent normal tissues (n = 409) was utilized to study PLA2G7 expression in prostate tissues. The samples were stained with previously validated PLA2G7 specific antibody, and the staining intensity was scored as presented in Figure 1A [9]. The results confirmed that PLA2G7 expression strongly associates with prostate cancer. PLA2G7 was expressed in 50.0% of the primary prostate tumor samples, whereas only 2.7% of the adjacent normal tissues showed any staining (Figure 1B-C and Supplemental Table S1). Importantly, the positive staining of PLA2G7 significantly correlated with high (≥ 7) Gleason score (Figure 1D and Supplemental Table S1). In accordance to the association of PLA2G7 expression and higher Gleason score, the results from Kaplan-Meier analysis suggested that PLA2G7 positivity associates with poor survival and more aggressive disease (Figure 1E).

### PLA2G7 silencing decreases the level of lysophosphatidylcholine

**Figure 2:**
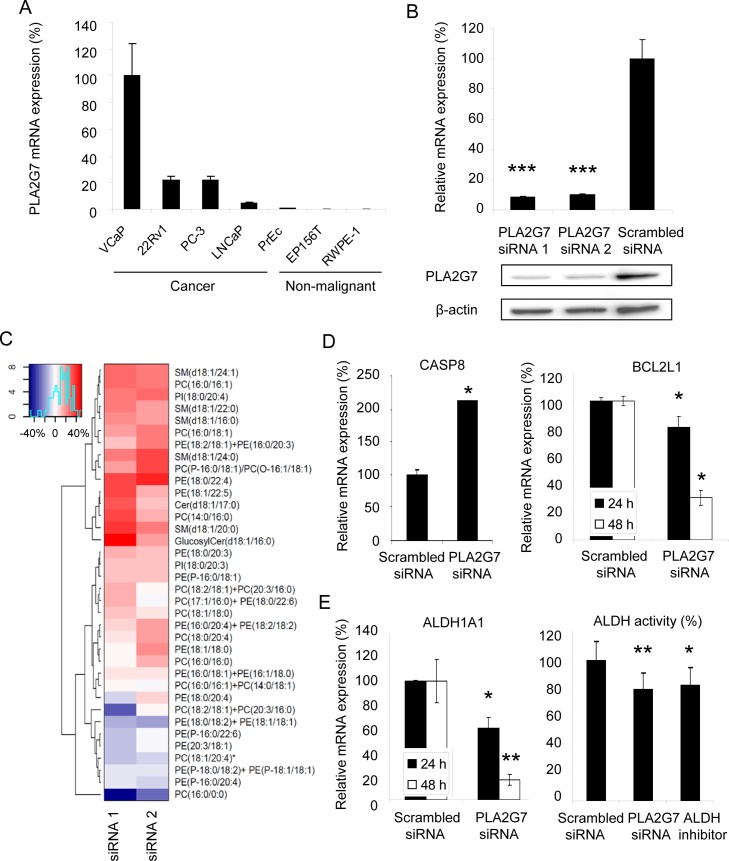
*PLA2G7* silencing decreases the level of lysophosphatidylcholine, induces apoptosis and reduces prostate tumorigenesis (A) PLA2G7 mRNA expression in cancerous and non-malignant prostate cell lines. (B) Validation of *PLA2G7* gene silencing in VCaP prostate cancer cells at mRNA and protein level. (C) Heatmap presentation of the cellular lipidomic changes in response to *PLA2G7* silencing in VCaP cells. The profiles were obtained at 48 h after transfection with two PLA2G7 siRNAs and compared to the respective scrambled siRNA control sample. The results are presented as the mean percentual change of two replicates in relation to the scrambled siRNA samples. Red color indicates upregulation and blue downregulation in response to *PLA2G7* silencing. PC, phosphatidylcholine; PE, phosphatidylethanolamine; PI, phosphatidylinositol; SM, sphingomyelin, Cer, ceramide. (D) The effect of *PLA2G7* silencing on the mRNA expression of pro-apoptotic *CASP8* at 24 h and anti-apoptotic *BCL2L1* at 24 h (left) and 48 h (right). (E) The effect of *PLA2G7* silencing on the mRNA expression of ALDH1A1 at 24 h (left) and 48 h (right), and the relative ALDH activity at 48 h. ALDH inhibitor DEAB was used as a positive control. Significant p-values in comparison to scrambled control are indicated.

Supporting the key role of altered lipid metabolism in prostate carcinogenesis, westernized corn oil containing diet has been shown to enhance cancer progression in mice, whereas thiazolidinediones have been reported to inhibit prostate cancer cell growth *in vitro* and *in vivo* [20, 21]. In order to reveal the lipidomic changes induced by PLA2G7 impairment, cellular lipidomic profiles were analyzed in ERG positive VCaP prostate cancer cells expressing PLA2G7 at high levels (Figure 2A). Target gene silencing was confirmed using qRT-PCR and western blot analysis (Figure 2B). Ultra Performance Liquid Chromatography - Mass Spectrometry (UPLC-MS) results indicate that the most prominent change in response to 48 h PLA2G7 silencing was a decrease in the cellular lysophosphatidylcholine (LPC, PC(16:0/0:0), 1-hexadecanoyl-*sn*-glycero-3-phosphocholine) level (Figure 2C and Supplemental Table S2). This is in agreement with the known function of PLA2G7 in cardiovascular diseases and previous results with PLA2G7 inhibitor treatment showing significant decrease in LPC levels in the arteries of pigs with induced diabetes and hypercholesterolemia [11].

### PLA2G7 silencing induces apoptosis

To get additional insights into the molecular mechanisms regulated by PLA2G7, genome-wide gene expression profiling was performed in *PLA2G7* impaired prostate cancer cells. The results indicated that cell-to-cell signaling and interaction, as well as cell death were the most significantly enriched biological processes in response to PLA2G7 silencing (Table 1 and Supplemental Table S3). Induction of cell death, validated by measuring pro-apoptotic CASP8 and anti-apoptotic BCL2L1 mRNA expression (Figure 2D), is in accordance to our previous results, since *PLA2G7* silencing was shown to induce caspase 3 and 7 activity in VCaP cells [9], indicating that *PLA2G7* silencing activates both intrinsic and extrinsic apoptotic pathways.

**Table 1:** The effect of *PLA2G7* silencing on VCaP gene expression profile The functional gene ontology and pathway annotations were analyzed for the sets of differentially expressed genes (logFC > 0.4 or < -0.4; FC > 1.32 or < 0.76) using Ingenuity Pathway Analysis Software.

**24 h**		
**Molecular and Cellular Functions**	n ^a^	p-value
Cell Cycle	28	5.53E-07 - 2.08E-02
Cell-To-Cell Signaling and Interaction	26	2.37E-05 - 1.92E-02
Cellular Development	19	3.77E-05 - 1.96E-02
Cell Morphology	20	3.92E-05 - 2.08E-02
Cell Death	41	7.98E-05 - 2.08E-02
**Canonical Pathways**	n ^a^	p-value
Rac Signaling	6	0.000353
TNFR1 Signaling	4	0.000805
Ephrin Receptor Signaling	7	0.00089
PAK Signaling	5	0.0012
Induction of Apoptosis by HIV1	4	0.00189
**48 h**		
**Molecular and Cellular Functions**	n ^a^	p-value
Cell-To-Cell Signaling and Interaction	16	1.52E-05 - 1.56E-02
Cell Death	24	2.00E-05 - 1.56E-02
Cellular Assembly and Organization	17	1.10E-04 - 1.17E-02
Cellular Function and Maintenance	13	1.10E-04 - 1.47E-02
Cellular Compromise	6	1.51E-04 - 1.17E-02
**Canonical Pathways**	n a	p-value
Integrin Signaling	5	0.000974
Valine. Leucine and Isoleucine Biosynthesis	2	0.00115
Estrogen Receptor Signaling	4	0.0016
Huntington's Disease Signaling	5	0.00191
Glucocorticoid Receptor Signaling	5	0.00323

**a The number of genes (up and down) regulated due to PLA2G7 silencing.**

### PLA2G7 silencing reduces tumorigenesis inducing aldehyde dehydrogenase activity

The presence of self-renewing and multi-potent cancer stem cells has been studied also in prostate cancer and signaling pathways implicated in the regulation of stem cell features such as neuroendocrine differentiation have been proposed as potential therapeutic targets in prostate cancer [22]. Interestingly, *PLA2G7* silencing reduced the expression of aldehyde dehydrogenase 1A1 (*ALDH1A1*) (Figure 2E), described as a prostate cancer stem cell marker and associated with poor prostate cancer outcome [23, 24]. Furthermore, *PLA2G7* silencing reduced aldehyde dehydrogenase (ALDH) activity to the same level as ALDH inhibitor (Figure 2E). High ALDH activity has been reported to identify tumor-initiating as well as metastasis-initiating prostate cancer cells [25]. Taken together, these results support the association of PLA2G7 expression with aggressive disease and suggest that PLA2G7 promotes tumorigenesis and metastasis via inducing ALDH activity.

### PLA2G7 silencing reduces cell adhesion and motility

**Figure 3:**
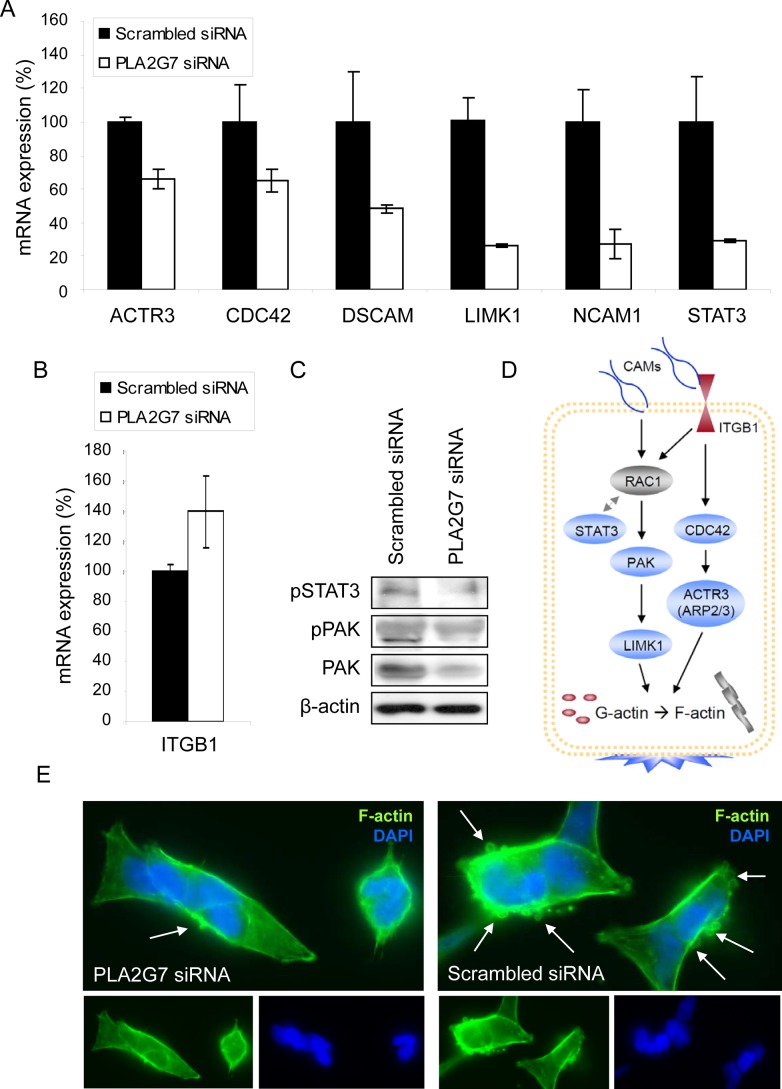
PLA2G7 affects the expression of multiple genes associated with cell adhesion and migration (A) The change in the relative mRNA expression of *ACTR3, CDC42*, *DSCAM*, *LIMK1, NCAM1*, and *STAT3* in response to 24 h siRNA transfection. (B) The effect of 48 h *PLA2G7* silencing on ITGB mRNA expression. (C) The change in pSTAT3, pPAK and PAK protein expression in response to 72 h *PLA2G7* silencing. β-actin is presented as an endogenous control. (D) Schematic illustration of the Rac1 and CDC42 signaling related gene products as a pathway. Blue color indicates downregulation and red upregulation in response to *PLA2G7* silencing. (E) Microscopic images (63 x) of PLA2G7 siRNA and scrambled siRNA transfected and LPC stimulated VCaP cells stained with phalloidin (F-actin, green; DAPI, blue) are shown. Arrows indicate the presence of cell protrusions.

The results from the genome-wide gene expression profiling indicated that cell motility and invasion related canonical pathways were among the top altered processes in response to *PLA2G7* silencing (Table 1 and Supplemental Table S3). Furthermore, the most outstanding change in the gene expression profile 48 h after *PLA2G7* knock-down was the increased mRNA expression of γ-actin (*ACTG1*, FC 3.84), suggesting possible dysregulation in F-actin polymerization. The altered mRNA expression of genes involved in cell adhesion (*DSCAM, ITGB1, NCAM1*), migration (*ACTR3, CDC42, LIMK1*) and metastasis (*STAT3*) as well as protein levels of PAK, pPAK and pSTAT3 were validated (Figure 3A-D). Supporting the putative role of PLA2G7 in migration and metastasis, F-actin staining of VCaP cells stimulated with LPC showed a clear decrease in the amount of cell protrusions after *PLA2G7* knock-down compared to scrambled siRNA treated cells (Figure 3E).

**Figure 4:**
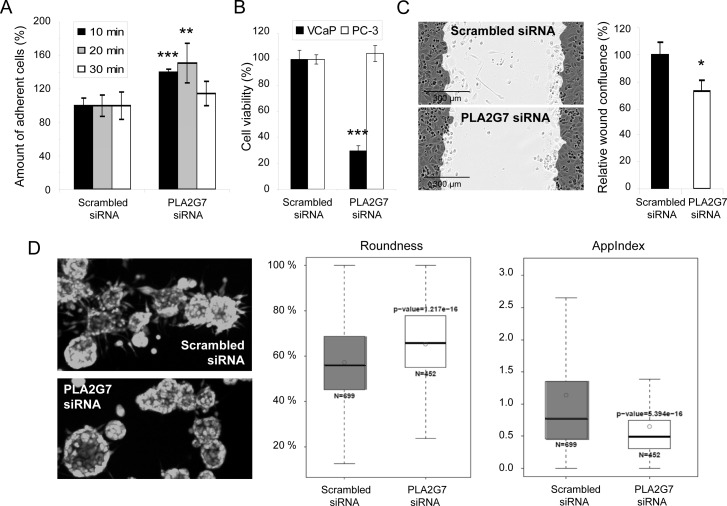
*PLA2G7* silencing decreases prostate cancer cell adhesion, migration and invasion (A) Fibronectin cell adhesion analysis. The relative amount of attached PI labeled cells is presented. (B) The effect of 72 h *PLA2G7* silencing on VCaP and PC-3 cell viability. (C) Wound healing assay with PC-3 cells following 72 h siRNA transfection. The results from 6 h time point after wound scratching are presented. (D) 3D cell invasion analysis. The spheroid roundness (%), and the relative index of invasive protrusions (AppIndex) in the 3D structures were measured from the microscopic (5 x) images. Significant p-values in comparison to scrambled control are indicated.

To validate the adhesion phenotype, cell attachment on fibronectin was monitored. The results indicated a significant acceleration in the adherence of cells to fibronectin in response to *PLA2G7* silencing (Figure 4A), mimicking the previously described *ERG* knock-down phenotype [3], and supporting the possibility that PLA2G7 is an important mediator of *ERG* oncogene in prostate cancer. Since VCaP cells do not migrate in wound healing experiment nor grow in 3D matrix [26], PC-3 cells, expressing *PLA2G7* (Figure 2A), were selected as a model to validate the functional effect of *PLA2G7* silencing on prostate cancer cell motility and invasion. Interestingly, as previously seen in LNCaP cells [9], *PLA2G7* silencing did not affect PC-3 cell viability (Figure 4B). However, the migratory capacity of PC-3 cells both in 2D and 3D cultures was decreased (Figure 4C-D) confirming the role of PLA2G7 in promoting prostate cancer cell migration and invasion.

### Statins potentiate the antiproliferative effect of PLA2G7 inhibition

Since statins are known to inhibit PLA2G7 in atherosclerotic plaques [19], their ability to reduce PLA2G7 expression and activity in VCaP prostate cancer cells was elucidated. However, although epidemiologic evidence supports the possible chemopreventive potential of statins in prostate cancer, previous studies with cultured prostate cancer cells have revealed that long exposure time (ad 5d) and micromolar concentrations of statins are needed to reduce cell growth *in vitro* [27-30].

**Figure 5:**
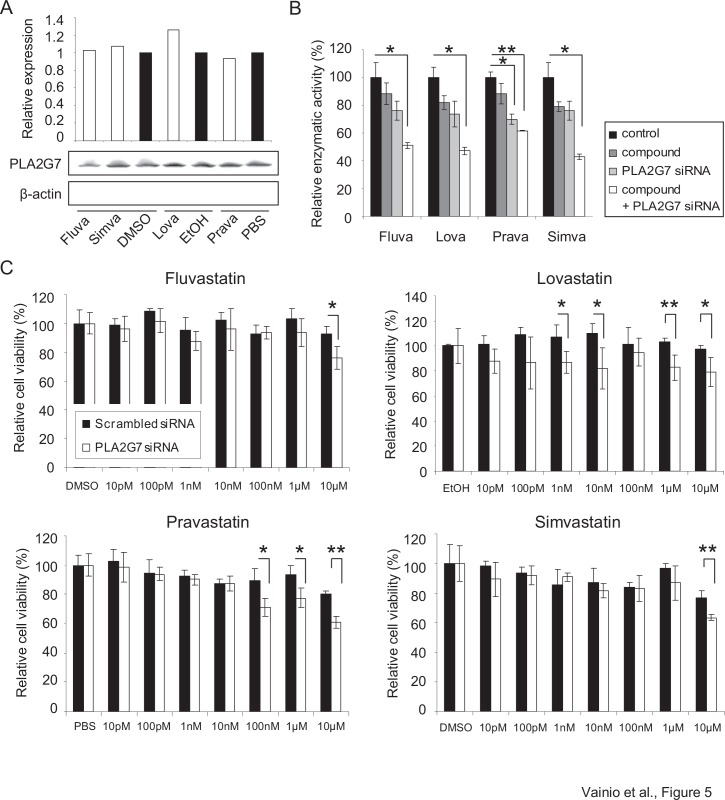
Statins decrease the enzymatic activity of PLA2G7 and act synergistically with *PLA2G7* silencing on prostate cancer cell viability (A) The effect of 48 h statin (10 μM) treatments on PLA2G7 protein expression in VCaP cells. The relative PLA2G7 (PLA2G7/β-actin) protein expression has been indicated with bars. (B) The effect of 10 μM statins alone and in combination with PLA2G7 silencing on PLA2G7 activity in VCaP cells. Significant p-values for individual treatments are given, compared with the scrambled control and diluent treated cells. (C) The relative effect of fluvastatin, lovastatin, pravastatin and simvastatin in combination with *PLA2G7* silencing on VCaP cell viability. Cell viability in diluent treated cells (scrambled and PLA2G7 siRNA transfected samples) was set as 100% to distinguish the synergism detected. Significant p-values for individual treatments are given, compared with the scrambled control.

The results from this study indicated that although PLA2G7 protein levels were not consistently affected by statins, the enzymatic activity of PLA2G7 was reduced by all four statins studied (Figure 5A-B). Furthermore, simvastatin, fluvastatin and lovastatin were able to inhibit PLA2G7 enzymatic activity synergistically with PLA2G7 siRNA. Due to these results connecting statins with PLA2G7 function also in prostate cancer, the effect of statins in combination with *PLA2G7* knock-down on VCaP cell viability was analyzed. Interestingly, even though the changes in cell viability were studied already after 48 h combinatorial treatment, the results indicated that statins synergistically reinforced the anti-proliferative effect of *PLA2G7* silencing (Figure 5C).

## DISCUSSION

In this study, we elucidated the potential of PLA2G7 as a biomarker and therapeutic drug target in prostate cancer management. By determining the expression level of PLA2G7 in malignant and healthy prostate samples from 453 prostate cancer patients, the biomarker potential of PLA2G7 and the size of the potential patient group benefiting from PLA2G7 inhibition therapy, was evaluated. The cellular responses to PLA2G7 inhibition were studied to understand the PLA2G7 driven biological and oncogenic processes in prostate cancer cells. Furthermore, as combinatorial therapeutic approaches may be required to obtain significant therapeutic progress, the ability of statins to potentiate the anti-proliferative effect of PLA2G7 impairment in prostate cancer cells was investigated.

Immunohistochemical staining results indicated that PLA2G7 is a potential prostate cancer biomarker present in approximately 50 percent of tumors in our cohort, and associating with high grade prostate tumors. Moreover, PLA2G7 impairment reduced aldehyde dehydrogenase activity, considered as a marker of prostate cancer stem cells as well as tumor- and metastasis-initiating prostate cancer cells [23-25] supporting the possibility that PLA2G7 expression may have prognostic significance. This hypothesis is further supported by our previous results demonstrating PLA2G7 protein expression in 70% of metastatic prostate tumors compared to the 50% positivity observed in the primary tumors [9]. Since PLA2G7 is induced by ERG and is highly expressed especially in the ERG positive prostate cancers, it is a putative biomarker for this subgroup of prostate cancers [4, 9]. The antiproliferative and pro-apoptotic effect of PLA2G7 impairment was seen in the ERG positive prostate cancer cells, indicating that ERG positive prostate cancer cells are dependent on PLA2G7 function [9]. However, PLA2G7 positivity is not restricted to ERG positive cancer cells since other oncogenic mutations have been recently shown to induce PLA2G7 expression [31].

PLA2G7 impairment modulated the levels of multiple lipids in prostate cancer cells, the most striking being the reduction in lysophosphatidylcholine (LPC). In addition to promoting cardiovascular diseases, LPC is known to increase the expression of cell-to-cell adhesion molecules on endothelial cells and to induce the migration and proliferation of smooth muscle cells [32, 33]. Furthermore, LPC has been linked to cancer cell migration and metastasis via promoting invadopodia formation in multiple cancer cell lines as well as migration of PC-3 prostate cancer cells [34, 35]. Our results support a similar role for LPC in prostate cancer cells since *PLA2G7* silencing followed by reduced LPC levels resulted in changes in multiple adhesion molecules, such as decrease in the expression of cell-to-cell adhesion molecules NCAM1 and DSCAM, and increase in extracellular matrix binding ITGB1. In addition to accelerated adhesion to fibronectin, *PLA2G7* silencing reduced cell migration and invasion in prostate cancer cell culture models. Furthermore, Rac1, CDC42 and STAT3 signaling, known to promote invadopodia formation and metastasis, were reduced by PLA2G7 impairment [36, 37]. STAT3 is also known to promote androgen-independent growth in cultured prostate cancer cells [38], giving additional support to the beneficial effect of PLA2G7 inhibition in prostate cancer management. The anti-migratory effect was not restricted to ERG positive prostate cancer cells supporting the rationale of PLA2G7 inhibition in the prevention and treatment of aggressive and metastatic tumors. Interestingly, *PLA2G7* silencing was recently shown to reduce xenograft growth of colon cells expressing mutant p53 and activated Ras [31], indicating that in addition to prostate cancers, PLA2G7 may have potential as a drug target in other cancer types as well.

In melanoma cells heat shock protein 90 (HSP90) inhibitors have been suggested to exert part of their antineoplastic effects by modulating phospholipase activity and related metabolic changes [39]. Furthermore, in cardiovascular diseases widely used and clinically well tolerated lipid-lowering statins are known to exert part of their beneficial effects via PLA2G7 inhibition [14, 18, 19]. This knowledge in combination with frequent expression and vital function of PLA2G7 in ERG positive prostate cancer cells provides a significant opportunity for drug repositioning. Epidemiologic evidence supports the possible chemopreventive potential of statins especially in advanced prostate cancer, and recently statins have been associated with better outcomes among men receiving radiotherapy for prostate cancer [27, 28, 40, 41]. Statins decrease androgen receptor protein expression and induce apoptosis and cell growth arrest in cultured prostate cancer cells, and suppress tumor growth in prostate cancer mice xenografts [29, 30, 42]. Here we show for the first time that statins reduce the enzymatic activity of PLA2G7 in prostate cancer cells. Furthermore, a synergistic anti-proliferative effect was observed in response to combinatorial treatment with PLA2G7 inhibition and statins in cultured prostate cancer cells.

**Figure 6:**
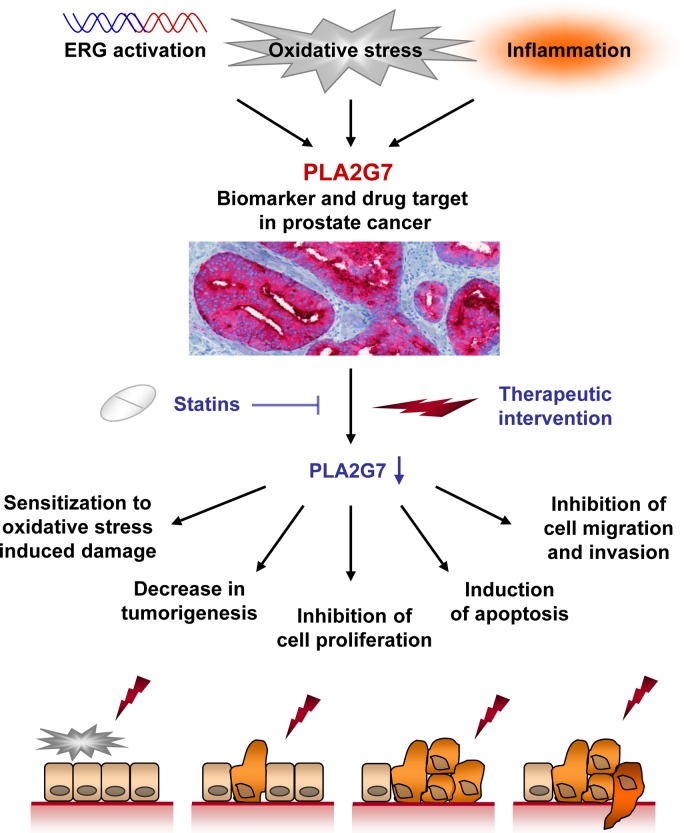
Schematic figure showing the putative potential of PLA2G7 impairment in prostate cancer management PLA2G7 expression and enzymatic function can be activated by e.g. ERG oncogene, inflammation or by substrates produced by oxidative stress [9, 43]. Our results suggest that PLA2G7 is a potential novel prognostic and therapeutic biomarker associating with aggressive disease. Therapeutic intervention impairing the expression or function of PLA2G7 induces multiple antineoplastic effects in cultured prostate cancer cells. PLA2G7 inhibition sensitizes prostate cancer cells to oxidative stress induced damage, decreases tumorigenetic potential and proliferation, as well as induces apoptosis and inhibits cancer cell migration. Moreover, clinically widely used statins inhibit the enzymatic activity of PLA2G7 and synergistically inhibit prostate cancer cell viability with RNAi induced PLA2G7 inhibition.

In conclusion, we propose PLA2G7 as a prognostic and theranostic biomarker, as well as a putative therapeutic target in prostate cancer (Figure 6). PLA2G7 promotes several oncogenic processes such as cell viability, migration and invasion in prostate cancer. Moreover, this is the first study connecting statin treatment with reduced PLA2G7 activity in prostate cancer cells, and presents a rationale for combining PLA2G7 inhibition with statins in prostate cancer management.

## METHODS

### Clinical prostate cancer samples

Samples from 453 prostate cancer patients treated with total prostatectomy between the years 1982 and 1998 at the Helsinki University Central Hospital, Finland, were included in this study. The allowance was obtained from the local ethical committee and all tissue samples were acquired and used according to contemporary regulatory guidelines. Median age of the patients at diagnosis was 63 (44 – 83) years. None of the patients had received adjuvant therapy before or immediately after the surgery. Histopathological features were re-viewed using the corresponding haematoxylin and eosin or herovici stained slides by two experienced pathologists (A.S. and S.N.), and clinical follow-up information was collected from patient files. Median tumour area (the percentage of tumour in all patient's histological sections) was 15%, ranging from 1% to 100%, and the median post-surgery follow-up time was 13.3 (11.3 – 25.0) years.

### Immunohistochemistry and statistical analysis of stainings

Tissue microarray (TMA) blocks were constructed using archival formalin-fixed paraffin-embedded (FFPE) prostatectomy blocks. Recipient blocks were predrilled with an automated TMA instrument (TMA Master, 3D Histech) and donor block cores (Ø 1.0 mm) were punched manually (Tissue-Tek® Quick-RayTM, Sakura Finetek). The TMA blocks consisted of four samples from each patient; two tissue cores from the area containing the most dominant Gleason grade pattern, one core out of the area with second most dominant Gleason grade pattern and one core from an adjacent benign glandular area. TMA blocks contained a total of 1,802 cores. All cancer cores were graded individually by experienced pathologists (A.S., S.N.) according to contemporary Gleason grading criteria.

Freshly cut TMA sections mounted on electrically charged glass slides (SuperFrost® Plus, Menzel-Gläser, Germany) were stained with a fully automatic immunohistochemical system Benchmark XT (Ventana Medical Systems, Illkirch, France) using a biotin-free multimer-based detection system (*ultra*View™ Universal Red, Ventana Medical Systems). The slides were pre-treated with standard cell conditioning using CC1 buffer and incubated for 60 minutes with the rabbit polyclonal primary antibody (1:150, Cayman Chemical). Hematoxylin II and Bluing Reagent (Ventana Medical Systems) were used as counterstains. Experiment included normal salivary gland as an external positive control tissue. Negative controls were performed by replacing the primary antibody with corresponding rabbit IgG.

The TMA slides were digitized with an automated whole slide scanner (Mirax Scan, Zeiss, Göttingen, Germany), using a 20 x objective (numerical aperture 0.75) and a Sony DFW-X710 camera with a 1024 x 768 pixel CCD sensor (Sony Corporation, Tokyo, Japan). The pixel resolution was 0.26 μm. The images were compressed to a wavelet file format (Enhanced Compressed Wavelet, ECW, ER Mapper, Erdas Inc, Atlanta, Georgia) with a conservative compression ratio of 1:5. The compressed virtual slides were uploaded to a web server (http://fimm.webmicroscope.net) running image server software (Image Web Server, Erdas Inc, Atlanta, Georgia).

The expression of PLA2G7 was evaluated by the pathologists (A.S., S.N., T.M.) blinded to the clinicopathological data at the time of scoring. Disease specific survival was defined as the time from diagnosis to the time of death due to prostate cancer. Patients who died of intercurrent causes were censored. Survival curves were calculated according to Kaplan-Meier and patient survival differences were analyzed using the log rank test. Gleason grading was available for 1,546 (85.8%) of the 1,802 TMA cores stained with PLA2G7 antibody and survival information was sufficient for 365 patients. In the survival analysis, a maximum score of each cancer cores of an individual patient was correlated with the clinical end-point.

### Cell culture

VCaP prostate cancer cells were received from Kenneth Pienta (University of Michigan, MI) or purchased from American Type Culture Collection (LGC Promochem AB, Borås, Sweden), and PC-3 cells were purchased from American Type Culture Collection (LGC Promochem AB). Both cell lines were grown in RPMI-1640 medium (Invitrogen, Carlsbad, CA). LNCaP cells were received from Dr. Marco Cecchini (University of Bern, Switzerland) and EP156T from Dr. Varda Rotter (Weizmann Institute of Science,Rehovot, Israel). The 22Rv1 cells were purchased from Deutsche Sammlung von Microorganismen und Zellkulturen GmbH (DSMZ, Braunschweig, Germany), RWPE-1 cells from American Type Culture Collection (LGC Promochem AB) and primary prostate epithelial cells (PrEc) from Lonza (Lonza Group Ltd, Basel, Switzerland).

Fluvastatin, lovastatin, pravastatin and simvastatin were purchased from Tocris (Tocris Bioscience, Ellisville, MO). Fluvastatin and simvastatin were diluted in DMSO, lovastatin in ethanol and pravastatin in PBS.

### Gene knock-down using RNA interference

Specific gene knock-downs using siRNA molecules (Qiagen GmbH, Hilden, Germany) targeting PLA2G7 (SI00072177, AAGGACTCTATTGATAGGGAA; SI00072184, TCCGTTGGTTGTACAGACTTA) were performed. AllStars Negative Control scrambled siRNA (Qiagen) was used as a negative control. The siRNAs were pipeted onto plates, followed by addition of the transfection agent (siLentFect Lipid Reagent, Bio-Rad Laboratories, Hercules, CA) and appropriate amount of cells. The PLA2G7 siRNAs were used either separately or as a pooled siRNA and the final siRNA concentration was 13 nM.

### Lipidomic profiling

VCaP cells were transfected in two replicates with two separate PLA2G7 siRNAs as well as scrambled control siRNA. After 48 h the cells were detached and washed two times with cold PBS and cell pellets were frozen to -80 °C. The cell pellets were homogenized with 50 μl PBS by using 3 grinding balls (Ø 3 and 5 mm) with a Retsch mixer mill MM400 homogenizer at 20 Hz for 2 min. From the homogenate, 5 μl was taken for the protein assay and 15 μl was used for lipid extraction (chloroform:methanol (2:1; 100 μl)) and spiked with an internal standard mixture of PC(17:0/0:0), MG(17:0/0:0/0:0)[rac], PG(17:0/17:0)[rac], Cer(d18:1/17:0), PS(17:0/17:0), PC(17:0/17:0), PA(17:0/17:0), PE(17:0/17:0), DG(17:0/17:0/0:0)[rac] and TG(17:0/17:0/17:0) at concentration levels of 0.1-0.2 μg / sample. The samples were vortexed for 2 min, incubated 30 min at RT and centrifuged at 7800 g for 3 min.

After extraction, the lower phase (60 μl) was separated and spiked with the labelled internal standard mixture containing PC(16:1-D3/0:0), PC(16:1/16:1-D6) and TG(16:0/16:0/16:0-13C3) at concentration level of 0.1 μg/sample. Lipid extracts were analysed on a Waters Q-Tof Premier mass spectrometer combined with an Acquity Ultra Performance LCTM (UPLCTM). The column used was an Acquity UPLCTM BEH C18 2.1 × 100 mm with 1.7 μm particles. The solvent system was A) ultrapure water (1% 1 M NH4Ac, 0.1% HCOOH) and B) LC/MS grade acetonitrile/isopropanol (1:1, 1% 1M NH4Ac, 0.1% HCOOH) and the gradient started from 65% A / 35% B, reached 80% B in 2 min, 100% B in 7 min and remained there for 7 min. The flow rate was 0.400 ml/min and the injected aliquot 2.0 μl (Acquity Sample Organizer, at 10 °C). Leucine enkephaline was used as the lock spray reference compounds. The data were collected at mass range of m/z 200-1,200 in negative ion mode with scan duration of 0.2 s. The data were processed using MZmine 2 software (http://mzmine.sourceforge.net/) and the lipid identification was based on an internal spectral library and tandem mass spectrometry. Protein content of the samples was determined from the PBS homogenate of the cells (5 μl) which was diluted further for Micro BCATM Protein Assay Kit (Pierce, Rockford, IL). Spectrophotometric determination was performed on a Multiskan EX instrument (Thermo Scientific, Vantaa, Finland), and the total protein content of the sample was used in the normalization of the data.

### Gene expression analysis with bead-arrays

Total RNA was extracted using RNeasy (Qiagen) and integrity of the RNA was monitored using Bioanalyzer 2100 (Agilent Technologies) according to manufacturer's instructions. Purified total RNA (500 ng) was amplified with the TotalPrep Kit (Ambion, Austin, TX) and the biotin labeled cRNA was hybridized to Sentrix HumanRef-12 Expression BeadChips (Illumina, San Diego, CA). The arrays were scanned with the BeadArray Reader (Illumina). The raw gene expression data were quantile-normalized and analyzed with the R / Bioconductor software [44]. Statistical analysis of differential gene expression was performed using the empirical Bayes statistics implemented in the eBayes function of the limma package [45]. Gene expression profiles of the PLA2G7 knock-down samples were compared to the respective control samples. The threshold for differential expression was q < 0.05 after the Benjamini-Hochberg multiple testing correction. The functional gene ontology and pathway annotations were analyzed for the sets of differentially expressed genes (logFC > 0.4 or < -0.4; FC > 1.32 or < 0.76) using Ingenuity Pathway Analysis Software (Ingenuity Systems Inc., Redwood City, CA, USA). Microarray data have been deposited in the ArrayExpress database (www.ebi.ac.uk/arrayexpress) under accession number E-TABM-1172.

### TaqMan quantitative reverse transcriptase PCR

RNA samples extracted with RNeasy Mini Kit (Qiagen), were reversely transcripted to cDNA (High Capacity cDNA Reverse Transcription Kit, Applied Biosystems) and PCR reaction samples were analyzed in 96-well or 384-well format. TaqMan quantitative reverse transcriptase PCR (qRT-PCR) analysis (Finnish DNA Microarray Centre, Centre for Biotechnology, University of Turku) was performed using ABI Prism 7900 (Applied Biosystems) and quantitation was carried out using the _ΔΔ_CT method with RQ manager 1.2 software (Applied Biosystems). At least two replicate samples were studied for detection of target mRNA expression and β-actin was used as an endogenous control. The primers and probes were designed and selected with the help of Universal ProbeLibrary Assay Design Center (Roche Diagnostics) (Supplemental Table S4).

### Western blot analysis

Whole-cell lysates were prepared using lysis buffer (62.5 mM Tris, 1% SDS, 5%, β-mercaptoethanol 10% glycerol, bromophenol blue). Antibodies used included, anti-pPAK (1:500, 2606S, Cell signaling technology, Danvers, MA), anti-PAK (1:1000, sc-881, Santa Cruz Biotechnology, Santa Cruz, CA), anti-pSTAT3 (1:500, sc-7993, Santa Cruz Biotechnology) and anti-PLA2G7 (1:500, Cayman Chemical) antibodies, as well as secondary ECL IgG HRP-linked (1:4000, Amersham Life Sciences, Fairfield, CT) and Alexa Fluor (1:4000, Molecular Probes, Invitrogen) antibodies. β-actin (1:5000, antibody from Sigma) was used as a loading control. The probed proteins were detected using enhanced chemiluminescence system (Amersham Life Sciences) or Odyssey Infrared Imaging System (LI-COR Biosciences, Lincoln, NE) according to the manufacturer's instructions. The obtained signals were densitometrically analyzed with GeneTools software (SynGene, Synoptics Ltd, Cambridge, UK).

### Determination of aldehyde dehydrogenase (ALDH) activity

The activity of ALDH was determined with Aldefluor reagent (Stemcell Technologies, Vancouver BC, Canada) according to manufacturer's instructions. Cells (2,000 / well) were plated and transfected as described in 384-well plates and incubated for 48 h. Medium was removed and cells washed with PBS, 10 μl of Aldefluor (Stemcell Technologies, Vancouver BC, Canada) or Aldefluor with DEAB (ALDH inhibitor diethylaminobenzaldehyde) was added to the cells and incubated at 37 °C for 30 minutes. Solutions were removed, cells washed, and 20 μl of assay buffer added into each well. The fluorometric signal was determined with Envision Multilabel Reader (PerkinElmer, Massachusetts, MA).

### Immunofluorescence staining

For the immunofluorescence staining, 10 μM lysophosphatidyl choline (LPC, 1-hexadecanoyl-sn-glycerol-3-phosphorylcholine; Cayman Chemical, Ann Arbor, MI) was added to the siRNA transfected cells at 24 h time point. Cells were fixed at 72 h time point with 4% paraformaldehyde (PFA) in PBS, permeabilized with 0.2% Triton X-100, and blocked with 3% BSA / PBS. Cells were stained with Alexa conjugated Phalloidin (1:100, Molecular Probes, Invitrogen), nuclei with Vectashield mounting medium (Vector Laboratories, Burlingame, CA) containing DAPI and images were taken with Zeiss Axiovert 200M fluorescence microscope (Carl Zeiss AG, Oberkochen, Germany).

### Cell viability assay

Prostate cancer cells were transfected on a 384-well plate and cell viability determined with CellTiter-Glo cell viability assay (Promega) according to the manufacturer's instructions. In compound-siRNA combinatorial cell viability assays a dilution series (10 pM – 10 μM) of statins were added to the cells 24 h after transfection and cell viability was determined following 48 h combinatorial treatment. The results were scanned with EnVision Multilabel platereader (PerkinElmer / Wallac).

### Cell adhesion assay

Plates (96 wells) were coated with fibronectin (5 μg / ml; CalbioChem, San Diego, CA) and blocked with 0.1% bovine serum albumin (BSA). VCaP cells were harvested 72 hours after siRNA transfection and trypsin was inactivated with 0.2% soybean trypsin inhibitor (Sigma). Cells were suspended in 0.5% BSA in serum free RPMI, seeded (10000 / well) on the plates, and allowed to adhere for 10, 20 and 30 minutes at 37 °C. After washing with PBS, cells were fixed (4% PFA, 10 minutes) and stained with propidium iodide (PI). The attached PI-stained cells were counted using Acumen Assay Explorer (TTP LabTech Ltd, Royston, UK).

### Wound healing assay

PC-3 prostate cancer cells were transfected with siRNAs on 96-well plates (Essen ImageLock, Essen Instruments, UK). After 72 h transfection, when cells reached confluency, a wound was scratched across each well (Wound Maker 96 Tool, Essen Instruments). Wound confluence was monitored with Incucyte Live-Cell Imaging System and software (Essen Instruments) and the amount of cell motility was determined at 6 h by comparing the mean relative wound density in each experiment.

### 3D cell culture

PC-3 cells were transfected with siRNAs 72 h prior to detaching cells from monolayer cultures. Uncoated Angiogenesis μ-slide (Ibidi Gmbh, Germany) wells were filled with 10 μl of Matrigel / culture medium (1:1; 50%) and polymerized at 37 °C for 1 h. Transfected cells (20,000 cells / ml density, ~ 1,000 cells / well) were plated on the slides and left to attach for 1–2 h at 37 °C before covering with a second layer of Matrigel / culture medium (1:4, 25%). Matrigel was allowed to polymerize overnight at 37 °C. Cell culture medium was changed every second day. After 8 days the 3D cultures were incubated for 30 min at 37 °C with Calcein AM live cell dye (Invitrogen). Confocal three-dimensional images were taken using Zeiss Axiovert 200 M with spinning disc confocal unit Yokogawa CSU22 and a Zeiss Plan-Neofluar 5× objective. Z-stacks were acquired with a step-size of 19 μm. Intensity projections were created using SlideBook 4.2.0.7 and NIH ImageJ (http://rsbweb.nih.gov/ij/), and further analyzed with VTT Acca software (sensitivity 15; threshold 1, structures less than 40 pixels in area / size filtered out). Box plots were visualized with R.

### PLA2G7 activity assay

VCaP cells were plated and transfected with siRNAs 24 hours before addition of 10 μM statins. After 48 h of combinatorial treatment the samples were lysed, managed and analyzed with EnVision Multilabel platereader (PerkinElmer / Wallac) according to the instructions of PLA2G7 (PAF acetylhydrolase) activity assay manufacturer (Cayman Chemical).

### Statistical analysis

The results are presented as the mean ± SD. Statistical analyses were performed using Student's t-test (*, *P* < 0.05; **, *P* < 0.01; ***, *P* < 0.001).

## Supplementary Tables

Supplemental Table S1

Supplemental Table S2

Supplemental Table S3

Supplemental Table S4

## References

[1] Jemal A, Bray F, Center MM, Ferlay J, Ward E, Forman D (2011). Global cancer statistics. CA Cancer J Clin.

[2] Tomlins SA, Rhodes DR, Perner S, Dhanasekaran SM, Mehra R, Sun XW, Varambally S, Cao X, Tchinda J, Kuefer R, Lee C, Montie JE, Shah RB, Pienta KJ, Rubin MA, Chinnaiyan AM (2005). Recurrent fusion of TMPRSS2 and ETS transcription factor genes in prostate cancer. Science.

[3] Gupta S, Iljin K, Sara H, Mpindi JP, Mirtti T, Vainio P, Rantala J, Alanen K, Nees M, Kallioniemi O (2010). FZD4 as a mediator of ERG oncogene-induced WNT signaling and epithelial-to-mesenchymal transition in human prostate cancer cells. Cancer Res.

[4] Iljin K, Wolf M, Edgren H, Gupta S, Kilpinen S, Skotheim RI, Peltola M, Smit F, Verhaegh G, Schalken J, Nees M, Kallioniemi O (2006). TMPRSS2 fusions with oncogenic ETS factors in prostate cancer involve unbalanced genomic rearrangements and are associated with HDAC1 and epigenetic reprogramming. Cancer Res.

[5] Sun C, Dobi A, Mohamed A, Li H, Thangapazham RL, Furusato B, Shaheduzzaman S, Tan SH, Vaidyanathan G, Whitman E, Hawksworth DJ, Chen Y, Nau M, Patel V, Vahey M, Gutkind JS (2008). TMPRSS2-ERG fusion, a common genomic alteration in prostate cancer activates C-MYC and abrogates prostate epithelial differentiation. Oncogene.

[6] Tomlins SA, Laxman B, Varambally S, Cao X, Yu J, Helgeson BE, Cao Q, Prensner JR, Rubin MA, Shah RB, Mehra R, Chinnaiyan AM (2008). Role of the TMPRSS2-ERG gene fusion in prostate cancer. Neoplasia.

[7] Zong Y, Xin L, Goldstein AS, Lawson DA, Teitell MA, Witte ON (2009). ETS family transcription factors collaborate with alternative signaling pathways to induce carcinoma from adult murine prostate cells. Proc Natl Acad Sci U S A.

[8] Hermans KG, van Marion R, van Dekken H, Jenster G, van Weerden WM, Trapman J (2006). TMPRSS2:ERG fusion by translocation or interstitial deletion is highly relevant in androgen-dependent prostate cancer, but is bypassed in late-stage androgen receptor-negative prostate cancer. Cancer Res.

[9] Vainio P, Gupta S, Ketola K, Mirtti T, Mpindi JP, Kohonen P, Fey V, Perälä M, Smit F, Verhaegh G, Schalken J, Alanen KA, Kallioniemi O, Iljin K (2011). Arachidonic acid pathway members PLA2G7, HPGD, EPHX2, and CYP4F8 identified as putative novel therapeutic targets in prostate cancer. Am J Pathol.

[10] Stafforini DM (2009). Biology of platelet-activating factor acetylhydrolase (PAF-AH, lipoprotein associated phospholipase A2). Cardiovasc Drugs Ther.

[11] Wilensky RL, Shi Y, Mohler ER, Hamamdzic D, Burgert ME, Li J, Postle A, Fenning RS, Bollinger JG, Hoffman BE, Pelchovitz DJ, Yang J, Mirabile RC, Webb CL, Zhang L, Zhang P (2008). Inhibition of lipoprotein-associated phospholipase A2 reduces complex coronary atherosclerotic plaque development. Nat Med.

[12] Zalewski A, Macphee C (2005). Role of lipoprotein-associated phospholipase A2 in atherosclerosis: biology, epidemiology, and possible therapeutic target. Arterioscler. Thromb Vasc Biol.

[13] May HT, Horne BD, Anderson JL, Wolfert RL, Muhlestein JB, Renlund DG, Clarke JL, Kolek MJ, Bair TL, Pearson RR, Sudhir K, Carlquist JF (2006). Lipoprotein-associated phospholipase A2 independently predicts the angiographic diagnosis of coronary artery disease and coronary death. Am Heart J.

[14] O'Donoghue M, Morrow DA, Sabatine MS, Murphy SA, McCabe CH, Cannon CP, Braunwald E (2006). Lipoprotein-associated phospholipase A2 and its association with cardiovascular outcomes in patients with acute coronary syndromes in the PROVE IT-TIMI 22 (PRavastatin Or atorVastatin Evaluation and Infection Therapy-Thrombolysis In Myocardial Infarction) trial. Circulation.

[15] Oei HH, van der Meer IM, Hofman A, Koudstaal PJ, Stijnen T, Breteler MM, Witteman JC (2005). Lipoprotein-associated phospholipase A2 activity is associated with risk of coronary heart disease and ischemic stroke: the Rotterdam Study. Circulation.

[16] Packard CJ, O'Reilly DS, Caslake MJ, McMahon AD, Ford I, Cooney J, Macphee CH, Suckling KE, Krishna M, Wilkinson FE, Rumley A, Lowe GD (2000). Lipoprotein-associated phospholipase A2 as an independent predictor of coronary heart disease. West of Scotland Coronary Prevention Study Group. N Engl J Med.

[17] Serruys PW, García-García HM, Buszman P, Erne P, Verheye S, Aschermann M, Duckers H, Bleie O, Dudek D, Bøtker HE, von Birgelen C, D'Amico D, Hutchinson T, Zambanini A, Mastik F, van Es GA (2008). Effects of the direct lipoprotein-associated phospholipase A(2) inhibitor darapladib on human coronary atherosclerotic plaque. Circulation.

[18] Racherla S, Arora R (2010). Utility of Lp-PLA2 in Lipid-Lowering Therapy. Am J Ther.

[19] Schaefer EJ, McNamara JR, Asztalos BF, Tayler T, Daly JA, Gleason JL, Seman LJ, Ferrari A, Rubenstein JJ (2005). Effects of atorvastatin versus other statins on fasting and postprandial C-reactive protein and lipoprotein-associated phospholipase A2 in patients with coronary heart disease versus control subjects. Am J Cardiol.

[20] Lyles BE, Akinyeke TO, Moss PE, Stewart LV (2009). Thiazolidinediones regulate expression of cell cycle proteins in human prostate cancer cells via PPARgamma-dependent and PPARgamma-independent pathways. Cell Cycle.

[21] Vissapragada S, Ghosh A, Ringer L, Salinas P, Brophy A, Peaceman D, Kallakury B, Banerjee PP, Fricke ST, Helfrich W, Lee YC, Pestell R, Scherer P, Tanowitz HB, Avantaggiati ML, Hilakivi-Clarke L (2010). Dietary n-3 polyunsaturated fatty acids fail to reduce prostate tumorigenesis in the PB-ErbB-2 x Pten(+/-) preclinical mouse model. Cell Cycle.

[22] Qi J, Pellecchia M, Ronai ZA (2010). The Siah2-HIF-FoxA2 axis in prostate cancer – new markers and therapeutic opportunities. Oncotarget.

[23] Li T, Su Y, Mei Y, Leng Q, Leng B, Liu Z, Stass SA, Jiang F (2010). ALDH1A1 is a marker for malignant prostate stem cells and predictor of prostate cancer patients' outcome. Lab Invest.

[24] Yu C, Yao Z, Dai J, Zhang H, Escara-Wilke J, Zhang X, Keller ET (2011). ALDH activity indicates increased tumorigenic cells, but not cancer stem cells, in prostate cancer cell lines. In Vivo.

[25] van den Hoogen C, van der Horst G, Cheung H, Buijs JT, Lippitt JM, Guzmán-Ramírez N, Hamdy FC, Eaton CL, Thalmann GN, Cecchini MG, Pelger RC, van der Pluijm G (2010). High aldehyde dehydrogenase activity identifies tumor-initiating and metastasis-initiating cells in human prostate cancer. Cancer Res.

[26] Härmä V, Virtanen J, Mäkelä R, Happonen A, Mpindi JP, Knuuttila M, Kohonen P, Lötjönen J, Kallioniemi O, Nees M (2010). A comprehensive panel of three-dimensional models for studies of prostate cancer growth, invasion and drug responses. PLoS One.

[27] Murtola TJ, Tammela TL, Määttänen L, Huhtala H, Platz EA, Ala-Opas M, Stenman UH, Auvinen A (2010). Prostate cancer and PSA among statin users in the Finnish prostate cancer screening trial. Int J Cancer.

[28] Platz EA, Leitzmann MF, Visvanathan K, Rimm EB, Stampfer MJ, Willett WC, Giovannucci E (2006). Statin drugs and risk of advanced prostate cancer. J Natl Cancer Inst.

[29] Hoque A, Chen H, Xu XC (2008). Statin induces apoptosis and cell growth arrest in prostate cancer cells. Cancer Epidemiol Biomarkers Prev.

[30] Yokomizo A, Shiota M, Kashiwagi E, Kuroiwa K, Tatsugami K, Inokuchi J, Takeuchi A, Naito S (2011). Statins reduce the androgen sensitivity and cell proliferation by decreasing the androgen receptor protein in prostate cancer cells. Prostate.

[31] McMurray HR, Sampson ER, Compitello G, Kinsey C, Newman L, Smith B, Chen SR, Klebanov L, Salzman P, Yakovlev A, Land H (2008). Synergistic response to oncogenic mutations defines gene class critical to cancer phenotype. Nature.

[32] Kohno M, Yokokawa K, Yasunari K, Minami M, Kano H, Hanehira T, Yoshikawa J (1998). Induction by lysophosphatidylcholine, a major phospholipid component of atherogenic lipoproteins, of human coronary artery smooth muscle cell migration. Circulation.

[33] Yokote K, Morisaki N, Zenibayashi M, Ueda S, Kanzaki T, Saito Y, Yoshida S (1993). The phospholipase-A2 reaction leads to increased monocyte adhesion of endothelial cells via the expression of adhesion molecules. Eur J Biochem.

[34] Harper K, Arsenault D, Boulay-Jean S, Lauzier A, Lucien F, Dubois CM (2010). Autotaxin promotes cancer invasion via the lysophosphatidic acid receptor 4: participation of the cyclic AMP/EPAC/Rac1 signaling pathway in invadopodia formation. Cancer Res.

[35] Monet M, Gkika D, Lehen'kyi V, Pourtier A, Vanden Abeele F, Bidaux G, Juvin V, Rassendren F, Humez S, Prevarsakaya N (2009). Lysophospholipids stimulate prostate cancer cell migration via TRPV2 channel activation. Biochim Biophys Acta.

[36] Abdulghani J, Gu L, Dagvadorj A, Lutz J, Leiby B, Bonuccelli G, Lisanti MP, Zellweger T, Alanen K, Mirtti T, Visakorpi T, Bubendorf L, Nevalainen MT (2008). Stat3 promotes metastatic progression of prostate cancer. Am J Pathol.

[37] Muralidharan-Chari V, Hoover H, Clancy J, Schweitzer J, Suckow MA, Schroeder V, Castellino FJ, Schorey JS, D'Souza-Schorey C (2009). ADP-ribosylation factor 6 regulates tumorigenic and invasive properties in vivo. Cancer Res.

[38] Lee SO, Lou W, Johnson CS, Trump DL, Gao AC (2004). Interleukin-6 protects LNCaP cells from apoptosis induced by androgen deprivation through the Stat3 pathway. Prostate.

[39] Beloueche-Babari M, Arunan V, Jackson LE, Perusinghe N, Sharp SY, Workman P, Leach MO (2010). Modulation of melanoma cell phospholipid metabolism in response to heat shock protein 90 inhibition. Oncotarget.

[40] Gutt R, Tonlaar N, Kunnavakkam R, Karrison T, Weichselbaum RR, Liauw SL (2010). Statin use and risk of prostate cancer recurrence in men treated with radiation therapy. J Clin Oncol.

[41] Kollmeier MA, Katz MS, Mak K, Yamada Y, Feder DJ, Zhang Z, Jia X, Shi W, Zelefsky MJ (2011). Improved biochemical outcomes with statin use in patients with high-risk localized prostate cancer treated with radiotherapy. Int J Radiat Oncol Biol Phys.

[42] Wang C, Tao W, Wang Y, Bikow J, Lu B, Keating A, Verma S, Parker TG, Han R, Wen XY (2010). Rosuvastatin, identified from a zebrafish chemical genetic screen for antiangiogenic compounds, suppresses the growth of prostate cancer. Eur Urol.

[43] Cao Y, Stafforini DM, Zimmerman GA, McIntyre TM, Prescott SM (1998). Expression of plasma platelet-activating factor acetylhydrolase is transcriptionally regulated by mediators of inflammation. J Biol Chem.

[44] Gentleman RC, Carey VJ, Bates DM, Bolstad B, Dettling M, Dudoit S, Ellis B, Gautier L, Ge Y, Gentry J, Hornik K, Hothorn T, Huber W, Iacus S, Irizarry R, Leisch F (2004). Bioconductor: open software development for computational biology and bioinformatics. Genome Biol.

[45] Smyth GK (2004). Linear models and empirical bayes methods for assessing differential expression in microarray experiments. Stat Appl Genet Mol Biol.

