# Supplementary material for: Phospholipase PLA2G7, associated with aggressive prostate cancer, promotes prostate cancer cell migration and invasion and is inhibited by statins
Source: Oncotarget. 2011 Dec 22;2(12):1176–90. doi: 10.18632/oncotarget.397 (PMC3282076; doi:10.18632/oncotarget.397)
Supplement: Supplemental Table S1 [file oncotarget-02-1176-s001.pdf]

Supplemental Table S1. Immunohistochemical staining results for PLA2G7 protein expression in prostate tissue microarray containing 409 non-malignant and 1137 cancerous tissue samples from 453 prostate cancer patients.

**Tissue samples**	**All**	**No staining**		**Positive staining**	
**Spot histology**	**n**	**n**	**%**	**n**	**%**
Benign	409	398	97.3	11	2.7
Malignant	1137	569	50	568	50
Gleason 6	444	270	60.8	174	39.2
Gleason 7	377	159	42.2	218	57.8
Gleason 3 + 4	219	94	42.9	125	57.1
Gleason 4 + 3	158	65	41.1	93	58.9
Gleason 8 - 10	316	140	44.3	176	55.7
Gleason 4 + 4	310	137	44.2	173	55.8
Gleason 4 + 5	5	2	40	3	60
Gleason 5 + 5	1	1	100	0	0
