# Supplementary material for: Phospholipase PLA2G7, associated with aggressive prostate cancer, promotes prostate cancer cell migration and invasion and is inhibited by statins
Source: Oncotarget. 2011 Dec 22;2(12):1176–90. doi: 10.18632/oncotarget.397 (PMC3282076; doi:10.18632/oncotarget.397)
Supplement: Supplemental Table S2 [file oncotarget-02-1176-s002.pdf]

Supplemental Table S2.
Cellular lipidomic profiles (UPLC-MS results) in response to 48 h PLA2G7 siRNA (2 separate siRNAs in 2 replicates) or Scrambled siRNA (2 replicates) trasfections in VCaP cells.

Name	m/z	RT	Num found	PLA2G7_siRNA1	PLA2G7_siRNA1	PLA2G7_siRNA2	PLA2G7_siRNA2	Scrambled	Scrambled
Cer(d18:1/17:0)	596,5264	386	78	0,093673	0,106893	0,089898	0,078027	0,065310	0,085319
GlucosylCer(d18:1/16:0)	698,5578	350	56	0,011260	0,024122	0,014349	0,012084	0,010846	0,011589
PC(14:0/16:0)	750,5315	339	50	0,074065	0,078604	0,075060	0,055610	0,053688	0,059247
PC(16:0/0:0)	480,3117	203	35	0,006939	0,008428	0,015219	0,008456	0,017381	0,015148
PC(16:0/16:0)	778,5621	371	62	0,063526	0,084494	0,091266	0,076074	0,073921	0,069718
PC(16:0/16:0)-d6	784,5985	369	49	0,037759	0,076875	0,062265	0,037924	0,045914	0,037692
PC(16:0/16:1)	688,5028	348	60	0,046067	0,071067	0,066552	0,048559	0,049923	0,042341
PC(16:0/16:1)+PC(14:0/18:1)	776,5441	340	62	0,326969	0,327804	0,375545	0,264418	0,316977	0,329186
PC(16:0/18:1)	804,5748	369	66	1,505105	2,211093	2,282173	1,636071	1,517704	1,613368
PC(17:1/16:0)+ PE(18:0/22:6)	790,5521	356	62	0,111771	0,191533	0,149112	0,110952	0,141100	0,120867
PC(18:0/20:4)	854,5946	360	9	0,008847	0,011487	0,012299	0,010358	0,010846	0,008278
PC(18:1/18:0)	832,6080	396	66	0,231102	0,360481	0,258136	0,296535	0,305288	0,221349
PC(18:1/20:4)*	852,5803	335	19	0,017694	0,029865	0,028698	0,020534	0,031634	0,022350
PC(18:2/18:1)+PC(20:3/16:0)	828,5797	356	8	0,016085	0,008031	0,009224	0,026758	0,021692	0,014065
PC(18:2/18:1)+PC(20:3/16:0)	828,5788	347	39	0,025457	0,036757	0,028698	0,026758	0,029826	0,024005
PC(P-16:0/18:1)/PC(O-16:1/18:1)	728,5606	416	14	0,059208	0,096291	0,086966	0,087575	0,070229	0,059686
PE(16:0/18:1)+PE(16:1/18.0)	716,5246	378	62	0,308216	0,365774	0,390015	0,271831	0,298059	0,339087
PE(16:0/20:4)+ PE(18:2/18:2)	738,5105	344	25	0,029899	0,038026	0,041209	0,031928	0,027159	0,035013
PE(18:0/18:2)+ PE(18:1/18:1)	742,5387	376	62	0,219303	0,311473	0,309411	0,216339	0,252999	0,378487
PE(18:0/20:3)	768,5518	389	13	0,058096	0,066366	0,077005	0,042636	0,041418	0,067138
PE(18:0/20:4)	766,5389	373	62	0,062293	0,081381	0,094645	0,074988	0,065730	0,088648
PE(18:0/22:4)	794,5672	389	8	0,012064	0,029865	0,022549	0,021579	0,018980	0,012417
PE(18:1/18:0)	744,5554	404	62	0,176337	0,248393	0,277672	0,227634	0,164338	0,252424
PE(18:1/22:5)	790,5435	341	48	0,048531	0,028716	0,043047	0,022442	0,028018	0,029695
PE(18:2/18:1)+PE(16:0/20:3)	740,5244	352	49	0,015961	0,021998	0,023458	0,019551	0,010882	0,023126
PE(20:3/18:1)	766,5397	357	30	0,011780	0,012635	0,013324	0,013811	0,013557	0,014072
PE(P-16:0/18:1)	700,5275	391	14	0,275301	0,376682	0,419446	0,248395	0,270085	0,320508
PE(P-16:0/20:4)	722,5143	356	14	0,164161	0,242366	0,221334	0,167148	0,229428	0,199847
PE(P-16:0/22:6)	746,5151	345	14	0,131378	0,136187	0,199058	0,107865	0,147924	0,159014
PE(P-18:0/18:2)+ PE(P-18:1/18:1)	726,5455	389	14	0,048182	0,061369	0,070070	0,037235	0,053149	0,061665
PI(18:0/20:3)	887,5678	356	61	0,063315	0,069312	0,068695	0,062662	0,061922	0,055866
PI(18:0/20:4)	885,5533	340	64	0,203449	0,194563	0,187849	0,222240	0,155338	0,166076
SM(d18:1/16:0)	747,5539	344	65	0,205650	0,248392	0,241957	0,203994	0,183742	0,189095
SM(d18:1/20:0)	803,6307	404	8	0,007238	0,013784	0,011274	0,007768	0,009501	0,005690
SM(d18:1/22:0)	831,6625	430	13	0,028954	0,036757	0,033823	0,028484	0,028018	0,024833
SM(d18:1/24:0)	859,6957	454	10	0,051816	0,074004	0,075453	0,058255	0,053615	0,045722
SM(d18:1/24:1)	857,6782	425	61	0,036412	0,054054	0,044072	0,044151	0,036374	0,033932
unidentified 1	554,3469	186	78	0,078125	0,108893	0,103498	0,090391	0,099481	0,099016
unidentified 10	692,6192	444	62	0,079378	0,086907	0,074406	0,081281	0,075766	0,057557

unidentified 100	797,5366	322	8	0,001609	0,002297	0,002050	0,002589	0,000904	0,000828
unidentified 101	773,5384	346	8	0,027675	0,039054	0,030477	0,027102	0,032759	0,028687
unidentified 102	818,6322	413	8	0,003217	0,005743	0,005125	0,006905	0,004519	0,003311
unidentified 103	740,5249	363	8	0,011260	0,010338	0,012299	0,012084	0,013333	0,007450
unidentified 104	815,5503	344	8	0,026541	0,036602	0,029509	0,020913	0,019884	0,025661
unidentified 105	859,5381	333	8	0,012868	0,021825	0,018449	0,020716	0,025167	0,013244
unidentified 106	666,0616	147	8	0,014650	0,009364	0,013861	0,012300	0,019210	0,020829
unidentified 107	745,5494	311	8	0,008043	0,009189	0,009525	0,011221	0,011068	0,008278
unidentified 11	764,5285	343	62	0,065778	0,086907	0,079190	0,072259	0,059153	0,067183
unidentified 12	861,5530	344	62	0,057629	0,084561	0,086850	0,054729	0,064691	0,065164
unidentified 13	582,5104	371	62	0,056744	0,081042	0,086966	0,088474	0,059508	0,065164
unidentified 14	802,5604	340	61	0,133252	0,157719	0,136415	0,127396	0,115596	0,106407
unidentified 15	788,5310	332	61	0,033779	0,063447	0,057065	0,057894	0,065614	0,057211
unidentified 16	687,5453	343	60	0,033779	0,045946	0,059753	0,035390	0,035249	0,038461
unidentified 17	770,5717	368	60	0,027100	0,041658	0,031479	0,033181	0,037042	0,030794
unidentified 18	872,5663	369	59	0,046971	0,074475	0,057660	0,048559	0,062845	0,048750
unidentified 19	858,6241	394	58	0,025737	0,052890	0,051380	0,043270	0,037960	0,038960
unidentified 2	543,3507	174	78	0,075015	0,083348	0,106895	0,076856	0,081924	0,097044
unidentified 20	646,6146	444	58	0,044424	0,052890	0,046146	0,046582	0,043462	0,035594
unidentified 21	744,5538	370	56	0,208839	0,257654	0,279239	0,196049	0,253824	0,189131
unidentified 22	716,5252	341	56	0,056744	0,057015	0,070997	0,037116	0,054538	0,048258
unidentified 23	810,5280	376	56	0,044162	0,062865	0,058706	0,037981	0,045593	0,043609
unidentified 24	744,5645	349	56	0,009416	0,010175	0,007143	0,009495	0,006327	0,005928
unidentified 25	792,5592	370	55	0,028149	0,044293	0,039973	0,034526	0,040693	0,031304
unidentified 26	748,5153	309	54	0,016890	0,029865	0,025623	0,020716	0,019884	0,020694
unidentified 27	826,6776	447	54	0,058920	0,066966	0,103241	0,069405	0,071152	0,058402
unidentified 28	480,3100	174	54	0,017734	0,017792	0,023148	0,021128	0,025614	0,019882
unidentified 29	762,5160	332	53	0,041571	0,065793	0,053473	0,068832	0,045308	0,044785
unidentified 3	749,5347	378	76	0,108138	0,149201	0,160951	0,122825	0,144402	0,119548
unidentified 30	742,5406	340	52	0,018463	0,021102	0,018160	0,014380	0,013116	0,014556
unidentified 31	778,5616	361	51	0,038615	0,041352	0,041837	0,040212	0,042539	0,039524
unidentified 32	824,6632	421	51	0,036211	0,056409	0,064986	0,048506	0,046735	0,038114
unidentified 33	891,6005	397	51	0,008847	0,014933	0,009599	0,009495	0,010846	0,010706
unidentified 34	694,6375	469	51	0,099029	0,111755	0,096515	0,134752	0,101115	0,100367
unidentified 35	770,5739	401	49	0,048531	0,057582	0,063674	0,053847	0,061922	0,042341
unidentified 36	774,5314	310	48	0,012868	0,017230	0,017424	0,012313	0,012653	0,009105
unidentified 37	807,5052	314	48	0,018498	0,026419	0,024598	0,021579	0,017173	0,019039
unidentified 38	722,4992	306	48	0,009651	0,019527	0,009639	0,010358	0,011750	0,009933
unidentified 39	833,5201	314	48	0,014432	0,027568	0,025623	0,027621	0,015365	0,013244
unidentified 4	483,3291	174	75	0,158418	0,202689	0,204649	0,129581	0,185809	0,187477
unidentified 40	854,6755	426	48	0,006434	0,009078	0,003075	0,007173	0,008134	0,005794
unidentified 41	808,6679	426	48	0,001609	0,007034	0,005125	0,003453	0,007231	0,004139
unidentified 42	508,4748	338	47	0,010455	0,018918	0,008199	0,009704	0,008134	0,007450

unidentified 43	682,5947	445	47	0,018498	0,025120	0,031773	0,028484	0,020788	0,017383
unidentified 44	690,5096	341	44	0,012064	0,011487	0,020499	0,009495	0,014461	0,010761
unidentified 45	816,5666	355	44	0,060851	0,066075	0,054520	0,052966	0,073921	0,058928
unidentified 46	782,4967	347	43	0,018498	0,018369	0,021422	0,015476	0,017173	0,021522
unidentified 47	534,4903	339	42	0,026541	0,025271	0,022549	0,013827	0,017529	0,019039
unidentified 48	784,5141	378	42	0,054792	0,069613	0,082197	0,058466	0,066195	0,051217
unidentified 49	809,5199	344	41	0,009558	0,009319	0,008199	0,007435	0,008134	0,007348
unidentified 5	830,5902	368	66	0,246106	0,359025	0,308607	0,326630	0,285842	0,291349
unidentified 50	664,5915	422	41	0,009651	0,018379	0,017424	0,015438	0,019620	0,013244
unidentified 51	812,6643	437	39	0,024128	0,039054	0,027673	0,035390	0,029826	0,019039
unidentified 52	686,4799	316	37	0,005630	0,005743	0,006082	0,004316	0,004519	0,005794
unidentified 53	806,5939	390	34	0,053459	0,082911	0,055566	0,046437	0,046231	0,048258
unidentified 54	465,3063	228	34	0,010794	0,005618	0,007610	0,005381	0,005489	0,014202
unidentified 55	690,6058	421	34	0,015281	0,027568	0,020499	0,018126	0,011750	0,014072
unidentified 56	786,5292	406	33	0,029629	0,047095	0,030748	0,029348	0,030730	0,033938
unidentified 57	889,5849	372	32	0,024932	0,036757	0,025623	0,032800	0,021692	0,026489
unidentified 58	762,5174	321	32	0,024932	0,044798	0,027673	0,022442	0,021692	0,027316
unidentified 59	688,4931	309	30	0,004021	0,006892	0,006150	0,006042	0,002711	0,002483
unidentified 6	835,5355	344	62	0,159069	0,186436	0,177110	0,158815	0,133726	0,134715
unidentified 60	586,4983	386	28	0,033779	0,065793	0,048240	0,044548	0,043749	0,039563
unidentified 61	540,3312	174	24	0,008557	0,010300	0,011958	0,010763	0,008233	0,014202
unidentified 62	618,5841	422	24	0,009651	0,011487	0,012299	0,012084	0,009038	0,007144
unidentified 63	812,5461	403	22	0,044424	0,059928	0,061846	0,071477	0,048077	0,045384
unidentified 64	478,2972	180	19	0,009252	0,009364	0,008697	0,008456	0,014636	0,008981
unidentified 65	666,6055	449	19	0,041139	0,045946	0,044072	0,047677	0,052692	0,033111
unidentified 66	664,6265	460	16	0,010455	0,016081	0,012299	0,012947	0,014461	0,011589
unidentified 67	510,0323	101	16	0,036046	0,061285	0,067143	0,039323	0,050528	0,040091
unidentified 68	818,5867	380	14	0,048531	0,055236	0,057660	0,046252	0,054538	0,041496
unidentified 69	776,5663	377	13	0,054558	0,075160	0,073360	0,054729	0,065157	0,049418
unidentified 7	863,5660	373	62	0,087357	0,120131	0,099105	0,110053	0,067342	0,084846
unidentified 70	775,5708	375	13	0,036255	0,055328	0,039928	0,043801	0,046520	0,055595
unidentified 71	772,5879	396	13	0,027345	0,042500	0,024598	0,033663	0,039770	0,026489
unidentified 72	856,6086	373	12	0,008043	0,012635	0,009571	0,009495	0,013557	0,009105
unidentified 73	758,5210	334	12	0,002413	0,002297	0,002050	0,001726	0,002711	0,001656
unidentified 74	748,5329	350	11	0,206214	0,252388	0,227552	0,217665	0,221380	0,206842
unidentified 75	764,5309	346	11	0,065778	0,086907	0,079190	0,072259	0,059153	0,067183
unidentified 76	911,5703	339	11	0,016890	0,024122	0,019474	0,018126	0,014501	0,015728
unidentified 77	773,5360	323	10	0,035390	0,057582	0,049286	0,034162	0,032538	0,036424
unidentified 78	772,5257	345	10	0,042363	0,061418	0,050866	0,041892	0,040195	0,035745
unidentified 79	883,5380	312	10	0,044424	0,057582	0,047980	0,049440	0,041616	0,038960
unidentified 8	714,5106	347	62	0,132242	0,211335	0,187393	0,130116	0,136296	0,132746
unidentified 80	750,5413	379	9	0,063315	0,086636	0,080686	0,074121	0,087861	0,070800
unidentified 81	774,5442	373	9	0,035748	0,059341	0,045492	0,048631	0,046627	0,041348

unidentified 82	662,6099	435	9	0,014477	0,013784	0,015374	0,010358	0,013557	0,011027
unidentified 83	826,6778	414	9	0,008526	0,006892	0,015374	0,008918	0,012653	0,007450
unidentified 84	752,5603	398	8	0,008043	0,014933	0,010249	0,009495	0,009942	0,008278
unidentified 85	799,5497	332	8	0,001609	0,004491	0,004100	0,003453	0,006327	0,004967
unidentified 86	801,5668	349	8	0,004021	0,004595	0,005125	0,003453	0,004519	0,004967
unidentified 87	724,5302	377	8	0,010455	0,013784	0,024598	0,017145	0,019884	0,014072
unidentified 88	915,6001	381	8	0,004021	0,006892	0,004100	0,006042	0,003615	0,004139
unidentified 89	825,5673	332	8	0,001609	0,001149	0,001025	0,000863	0,001808	0,001656
unidentified 9	536,5049	371	62	0,115225	0,165077	0,169768	0,153993	0,140096	0,108954
unidentified 90	754,5804	411	8	0,010455	0,014933	0,016399	0,020750	0,010846	0,014900
unidentified 91	788,5803	382	8	0,022520	0,023664	0,037923	0,026758	0,022596	0,024005
unidentified 92	796,5786	410	8	0,016085	0,025271	0,024598	0,018126	0,022596	0,024833
unidentified 93	775,5524	351	8	0,018498	0,026299	0,017270	0,016400	0,018980	0,016555
unidentified 94	780,5934	422	8	0,003217	0,003446	0,003075	0,002589	0,003588	0,003311
unidentified 95	827,5851	351	8	0,000804	0,002297	0,001025	0,001726	0,001808	0,001656
unidentified 96	778,5762	398	8	0,008822	0,010338	0,010249	0,012947	0,006524	0,006622
unidentified 97	816,6149	385	8	0,005630	0,011487	0,005125	0,006905	0,006327	0,004967
unidentified 98	798,6183	426	8	0,067589	0,064620	0,055566	0,067951	0,056384	0,054859
unidentified 99	829,6003	376	8	0,000804	0,001149	0,002050	0,000863	0,001808	0,001656
