# Supplementary material for: Phospholipase PLA2G7, associated with aggressive prostate cancer, promotes prostate cancer cell migration and invasion and is inhibited by statins
Source: Oncotarget. 2011 Dec 22;2(12):1176–90. doi: 10.18632/oncotarget.397 (PMC3282076; doi:10.18632/oncotarget.397)
Supplement: Supplemental Table S3 [file oncotarget-02-1176-s003.pdf]

Supplemental Table S3. The genes differentially expressed (FC > 1.32 or < 0.76) due to *PLA2G7* silencing and enriched in gene ontology and pathway annotations.

**24 h**	**Upregulated**	**n**	**Downregulated**	**n**
**Molecular and Cellular Functions**				
Cell Cycle	*ARNTL2, ATRX, EIF4G2, GNL3, IL6R, ITGB1, MAD2L1BP, MCM7, PAK2, RAN, UBA3, ZBTB10*	12	*ALOX15B, BAX, CDC42, CDKN2D, CENPE, FCGR2B, GMNN, LIMK1, NEDD9, NEK2, RAD21, SACM1L, SLC27A4, SNX33, STAT3, STK11*	16
Cell-To-Cell Signaling and Interaction	*CALD1, CASP8, GNAQ, IL6R, ITGB1, MAGI1, MUTYH, NET1, OGT, PAK2, TJP2*	11	*ADAM2, ALDH1A1, ALOX15B, BAX, C4BPA, CDC42, COL17A1, DSCAM, FCGR2B, GNPNAT1, LSP1, NCAM1, NEDD9, OXT, STAT3*	15
Cellular Development	*CASP8, DDX4, GNAQ, GNL3, IL6R, ITGB1, NCKAP1*	7	*BAX, CDC42, CDKN2D, GMNN, HIST1H1C, MAP2K5, MAST2, MTPN, NCAM1, OXT, STAT3, STK11*	12
Cell Morphology	*AMD1, CALD1, CASP8, GNAQ, IL6R, ITGB1, PAK2, SYTL2, TMPO*	9	*BAX, CDC42, HMGB2, LIMK1, LSP1, NCAM1, NEK2, PLN, RAD21, STAT3, STK11*	11
Cell Death	*ATRX, BIRC2, CASP8, DDX4, EIF4G2, GNAQ, GNL3, IL6R, ITGB1, NCKAP1, NOBOX, PAK2, PMEPA1, SON, TIA1, TJP2, UBA3, ZBTB10*	18	*ALDH1A1, ALOX15B, ATP2A1, BAX, CDC42, CDKN2D, FAM162A, FCGR2B, GMNN, GNPNAT1, HIST1H1C, HMGB2, HSPA4, LSP1, MAP2K5, MTPN, NCAM1, NEDD9, NEK2, RAD21, SNX33, STAT3, STK11*	23
**Canonical Pathways**				
Rac Signaling	*ITGB1, NCKAP1, PAK2*	3	*ACTR3, CDC42, LIMK1*	3
TNFR1 Signaling	*BIRC2, CASP8, PAK2*	3	*CDC42*	1
Ephrin Receptor Signaling	*GNAQ, ITGB1, PAK2*	3	*ACTR3, CDC42, LIMK1, STAT3*	4
PAK Signaling	*ITGB1, PAK2*	2	*CDC42, DSCAM, LIMK1*	3
Induction of Apoptosis by HIV1	*BIRC2, CASP8*	2	*BAX, SLC25A13*	2
**48 h**	**Upregulated**	**n**	**Downregulated**	
**Molecular and Cellular Functions**				
Cell-To-Cell Signaling and Interaction	*AR, CCNC, CD46, GGT1, HSPD1, ITGB1, KIF5B, NCL, NET1, PPP3R1, PRKDC, THBS1*	12	*BCL2L1, IGFBP3, NCOR2, STX1A*	4
Cell Death	*ALDOA, AR, CD46, CLK3, DHX9, EHF, EIF4G2, FZD3, GGT1, HSPD1, ITGB1, NCL, NSF, PPP3R1, PRKDC, RHOT1, SIM2, THBS1*	18	*BCL2L1, IGFBP3, NCOR2, NDUFAB1, POLR2A, STX1A*	6
Cellular Assembly and Organization	*ACTG1, AR, CD46, ITGB1, KIF5B, NET1, NSF, PICALM, PRKDC, PSMC4, SPTBN1, THBS1*	12	*BCL2L1, NFIX, SPOCK2, STX1A, WDR5*	5
Cellular Function and Maintenance	*ACTG1, AR, HSPD1, ITGB1, NSF, PICALM, PPP3R1, PRKDC, RHOT1, TCEA1, THBS1, TWSG1*	12	*BCL2L1*	1
Cellular Compromise	*ITGB1, PRKDC, SPTBN1, THBS1*	4	*BCL2L1, NDUFAB1*	2
**Canonical Pathways**				
Integrin Signaling	*ACTG1, ARF4, ITGB1, RHOT1*	4	*ARPC3*	1
Valine, Leucine and Isoleucine Biosynthesis	*IARS*	1	*VARS*	1
Estrogen Receptor Signaling	*CCNC, PRKDC*	2	*NCOR2, POLR2A*	2
Huntington's Disease Signaling	*NSF*	1	*BCL2L1, NCOR2, POLR2A, STX1A*	4
Glucocorticoid Receptor Signaling	*AR, PPP3R1*	2	*BCL2L1, NCOR2, POLR2A*	3
