# Supplementary material for: Phospholipase PLA2G7, associated with aggressive prostate cancer, promotes prostate cancer cell migration and invasion and is inhibited by statins
Source: Oncotarget. 2011 Dec 22;2(12):1176–90. doi: 10.18632/oncotarget.397 (PMC3282076; doi:10.18632/oncotarget.397)
Supplement: Supplemental Table S4 [file oncotarget-02-1176-s004.pdf]

**Supplemental Table S4.** Probes and primers utilized in the TaqMan qRT-PCR analysis.

| **Gene** | **Probe #** | **Forward primer** | **Reverse primer** |
|---|---|---|---|
| *ACTR3* | 9 | gaaaggtgttgatgacctagacttc | actataccatggcggattgg |
| *ALDH1A1* | 88 | gcaactgaggaggagctctg | gtcttgcggccttcactg |
| *BCL2L1* | 10 | gctgagttaccggcatcc | ttctgaagggagagaaagagattc |
| *CASP8* | 40 | gtctgtgcccaaatcaacaa | caaggctgctgcttctctct |
| *CDC42* | 22 | catcggaatatgtaccgactgtt | tgcagtatcaaaaagtccaagagta |
| *DSCAM* | 9 | aacctcatggacggagagc | agctccagtgaaggctgtgt |
| *ITGB1* | 65 | cgatgccatcatgcaagt | acaccagcagccgtgtaac |
| *LIMK1* | 36 | ggggcatcatcaagagca | tgtccttggcaaagctcact |
| *NCAM1* | 20 | taccgcggcaagaacatc | ccacctgcagagaaactgc |
| *PLA2G7* | 63 | tggctctaccttagaaccctga | ttttgctctttgccgtacct |
| *STAT3* | 14 | cccttggattgagagtcaaga | aagcggctatactgctggtc |
